# Functional treatment versus plaster for simple elbow dislocations (FuncSiE): a randomized trial

**DOI:** 10.1186/1471-2474-11-263

**Published:** 2010-11-12

**Authors:** Jeroen de Haan, Dennis den Hartog, Wim E Tuinebreijer, Gijs IT Iordens, Roelf S Breederveld, Maarten WGA Bronkhorst, Milko MM Bruijninckx, Mark R De Vries, Boudewijn J Dwars, Denise Eygendaal, Robert Haverlag, Sven AG Meylaerts, Jan-Willem R Mulder, Kees J Ponsen, W Herbert Roerdink, Gert R Roukema, Inger B Schipper, Michel A Schouten, Jan Bernard Sintenie, Senail Sivro, Johan GH Van den Brand, Hub GWM Van der Meulen, Tom PH Van Thiel, Arie B Van Vugt, Egbert JMM Verleisdonk, Jos PAM Vroemen, Marco Waleboer, W Jaap Willems, Suzanne Polinder, Peter Patka, Esther MM van Lieshout, Niels WL Schep

**Affiliations:** 1 Department of Surgery-Traumatology, Westfriesgasthuis, P.O. Box 600, 1620 AR Hoorn, The Netherlands; 2 Department of Surgery-Traumatology, Erasmus MC, University Medical Center Rotterdam, P.O. Box 2040, 3000 CA Rotterdam, The Netherlands; 3 Department of Surgery-Traumatology, Red Cross Hospital, P.O. Box 1074, 1940 EB Beverwijk, The Netherlands; 4 Department of Surgery-Traumatology, Bronovo Hospital, P.O. Box 96900, 2509 JH The Hague, The Netherlands; 5 Department of Surgery-Traumatology, IJsselland Hospital, P.O. Box 690, 2900 AR Capelle a/d IJssel, The Netherlands; 6 Department of Surgery-Traumatology, Reinier de Graaf Gasthuis, P.O. Box 5011, 2600 GA Delft, The Netherlands; 7 Department of Surgery-Traumatology, Slotervaart Hospital, P.O. Box 90440, 1006 BK Amsterdam, The Netherlands; 8 Department of Orthopaedics, Amphia Hospital, P.O. Box 90158, 4800 RK Breda, The Netherlands; 9 Department of Surgery-Traumatology, Onze Lieve Vrouwe Gasthuis, P.O. Box 95500, 1090 HM Amsterdam, The Netherlands; 10 Department of Surgery-Traumatology, Medical Center Haaglanden, P.O. Box 432, 2501 CK 's Gravenhage, The Netherlands; 11 Department of Surgery-Traumatology, Zaans Medical Center, P.O. Box 210, 1500 EE Zaandam, The Netherlands; 12 Department of Surgery-Traumatology, Academic Medical Center, P.O. Box 22660, 1100 DD Amsterdam, The Netherlands; 13 Department of Surgery-Traumatology, Deventer Hospital, P.O. Box 5001, 7400 GC Deventer, The Netherlands; 14 Department of Surgery-Traumatology, Maasstad Hospital, P.O. Box 9100, 3007 AC Rotterdam, The Netherlands; 15 Department of Surgery-Traumatology, Leiden University Medical Center, P.O. Box 9600, 2300 RC Leiden, The Netherlands; 16 Department of Surgery-Traumatology, Hospital Rivierenland, P.O. Box 6024, 4000 HA Tiel, The Netherlands; 17 Department of Surgery-Traumatology, Elkerliek Hospital, P.O. Box 98, 5700 AB Helmond, The Netherlands; 18 Department of Surgery-Traumatology, Flevo Hospital, P.O. Box 3005, 1300 EG Almere, The Netherlands; 19 Department of Surgery-Traumatology, Medical Center Alkmaar, P.O. Box 501, 1800 AM Alkmaar, The Netherlands; 20 Department of Surgery-Traumatology, Haga Hospital, P.O. Box 40551, 2504 LN 's Gravenhage, The Netherlands; 21 Department of Surgery-Traumatology, Hospital Queen Beatrix, P.O. Box 9005, 7100 GG Winterswijk, The Netherlands; 22 Department of Surgery-Traumatology, Medical Spectrum Twente, P.O. Box 50.000, 7500 KA Enschede, The Netherlands; 23 Department of Surgery-Traumatology, Diakonessenhuis, P.O. Box 80250, 3508 TG Utrecht, The Netherlands; 24 Department of Surgery-Traumatology, Amphia Hospital, P.O. Box 90158, 4800 RK Breda, The Netherlands; 25 Department of Surgery-Traumatology, Admiraal de Ruyter Hospital, P.O. Box 106, 4460 BB Goes, The Netherlands; 26 Department of Orthpaedic Surgery, Onze Lieve Vrouwe Gasthuis, P.O. Box 95500, 1090 HM Amsterdam, The Netherlands; 27 Department of Public Health, Erasmus MC, University Medical Center Rotterdam, P.O. Box 2040, 3000 CA Rotterdam, The Netherlands

## Abstract

**Background:**

Elbow dislocations can be classified as simple or complex. Simple dislocations are characterized by the absence of fractures, while complex dislocations are associated with fractures. After reduction of a simple dislocation, treatment options include immobilization in a static plaster for different periods of time or so-called functional treatment. Functional treatment is characterized by early active motion within the limits of pain with or without the use of a sling or hinged brace. Theoretically, functional treatment should prevent stiffness without introducing increased joint instability. The primary aim of this randomized controlled trial is to compare early functional treatment versus plaster immobilization following simple dislocations of the elbow.

**Methods/Design:**

The design of the study will be a multicenter randomized controlled trial of 100 patients who have sustained a simple elbow dislocation. After reduction of the dislocation, patients are randomized between a pressure bandage for 5-7 days and early functional treatment or a plaster in 90 degrees flexion, neutral position for pro-supination for a period of three weeks. In the functional group, treatment is started with early active motion within the limits of pain. Function, pain, and radiographic recovery will be evaluated at regular intervals over the subsequent 12 months. The primary outcome measure is the *Quick *Disabilities of the Arm, Shoulder, and Hand score. The secondary outcome measures are the Mayo Elbow Performance Index, Oxford elbow score, pain level at both sides, range of motion of the elbow joint at both sides, rate of secondary interventions and complication rates in both groups (secondary dislocation, instability, relaxation), health-related quality of life (Short-Form 36 and EuroQol-5D), radiographic appearance of the elbow joint (degenerative changes and heterotopic ossifications), costs, and cost-effectiveness.

**Discussion:**

The successful completion of this trial will provide evidence on the effectiveness of a functional treatment for the management of simple elbow dislocations.

**Trial Registration:**

The trial is registered at the Netherlands Trial Register (NTR2025).

## Background

The elbow joint is the second most commonly dislocated joint in adults. The annual incidence of elbow dislocations in children and adults is 6.1 per 100,000 [1]. Elbow dislocations are classified as simple or complex [2]. Simple dislocations are dislocations without fractures. Complex dislocations are associated with (avulsion) fractures of the distal humerus, radial head, ulna, or coronoid process. Conn et al. observed 414 injuries of the elbow, which included 58 elbow dislocations in both children and adults [3]. In 51% of these patients, the dislocations were of the simple type. Josefsson et al. observed 24 simple elbow dislocations in 52 patients (46%) who were 16 years old and older [4].

Elbow dislocations can also be classified by the direction of their displacement, i.e., posterior or anterior. Posterior dislocations can be subdivided into medial and lateral dislocations. Anterior dislocations are very rare. In the study by Conn et al., 96% of the dislocations were of the posterior or lateral type [3]. Moreover, Josefsson et al. observed no anterior dislocations in 52 elbow dislocations [4].

Different treatment modalities can be applied following reduction, including plaster immobilization, surgical treatment of ruptured collateral ligaments, functional treatment, or combinations. There is little available literature about treatment of elbow dislocations. One randomized controlled trial (RCT) was identified in which suture repair of the collateral ligaments was compared with conservative treatment with plaster [5]. No differences were found for loss of extension and flexion after more than one year, although a trend was found for enhanced flexion at five and ten weeks for the plaster group. However, this study lacked power, with a sample size of only 14 patients in each arm. When comparing functional treatment versus plaster immobilization, only one RCT was retrieved from the literature [6]. Extension and flexion of the elbow did not differ between the groups after one year. Nevertheless, a difference in elbow extension was observed at three months, favoring the patients treated functionally. Furthermore, when two observational studies were pooled comparing functional treatment with plaster immobilization, functional treatment showed a statistically significant better result for pain and range of motion (ROM) [7,8].

Three observational studies comparing different periods of plaster immobilization after reduction showed a larger ROM after shorter immobilization, but this finding was statistically significant in only one study [9-11]. Moreover, these studies may be confounded by the severity of the injury, as worse cases were probably immobilized longer.

An important question following reduction of simple elbow dislocations is whether or not the elbow is stable. Signs of instability are redislocation, a positive pivot shift test, positive valgus and varus stress testing, and radiographic incongruence. In the studies described above, stability testing was either not performed, or the tests differed between the studies. In these eight studies, only one recurrent dislocation after plaster treatment was mentioned [7] (i.e., one recurrence in 342 patients (0.3%)), and signs of gross instability were not mentioned. Therefore, we conclude that the majority of the patients included in these studies had simple dislocations, which remained stable after reduction. For this type of dislocation, literature suggests that plaster immobilization for more than two weeks following reduction may lead to limited ROM [12,13]. Therefore some authors state that early functional treatment should be the treatment of choice. Functional treatment is defined as early active movements within the limits of pain with or without the use of a sling or a hinged brace [6-8].

A recent electronic survey of 90 trauma surgeons in the Netherlands revealed that 60% of the patients with a simple elbow dislocation were generally treated with plaster immobilization for three weeks or longer [14].

The primary objective of this study is to compare the *Quick-*DASH (Disabilities of the Arm, Shoulder, and Hand) questionnaire scores after functional treatment versus plaster immobilization in adult patients who sustained a simple elbow dislocation. Secondary aims are to examine the effect of functional treatment versus plaster immobilization on functional outcome (Mayo Elbow Performance Index (MEPI) and Oxford elbow score), the level of pain (Visual Analog Scale (VAS)), ROM, the rate of secondary interventions and complications, health-related quality of life (Short Form-36 (SF-36) and EuroQol-5D (EQ-5D)), costs, and cost-effectiveness in these patients.

## Methods/Design

### Study design

The FuncSiE trial will follow a multicenter, randomized controlled trial design. Twenty-five centers in the Netherlands will participate. The study started August 26, 2009.

### Recruitment and consent

Eligible patients presenting to the emergency department (ED) with a simple elbow dislocation will be informed about the trial at the ED after reduction of the dislocated elbow. They will receive written information and a consent form from the attending physician, the clinical investigator or a research assistant. After providing informed consent, eligible patients will be randomized within one week. Participants will be allocated to one of two treatment arms using a web-based randomization program that will be available 24 hours a day. Variable block randomization will be accomplished via a trial website. Allocation will be at random.

It is not possible to blind surgeons and patients for the allocated treatment. In order to reduce bias, an independent researcher without knowledge of the prescribed treatment will perform follow-up measurements. In addition, radiographs will be blinded and evaluated in duplicate, and analysis will be done in a blinded fashion.

### Study population

All persons aged 18 years or older presenting with a simple elbow dislocation at the Emergency Departments of the participating clinics are eligible for inclusion.

Patients meeting the following inclusion criteria are eligible for enrolment:

1. Adult men or women aged 18 years and older (with no upper age limit)

2. A simple dislocation of the elbow (i.e., without associated fracture) that can be reduced by closed means. Presence of a dislocation and absence of fracture(s) will be confirmed by a plain X-ray

3. Provision of informed consent by patient

If any of the following criteria applies, patients will be excluded:

1. Polytraumatized patients

2. Patients with complex, pathological, recurrent or open dislocations

3. Additional traumatic injuries of the affected arm

4. Patients undergoing surgical repair of collateral ligaments of the dislocated elbow joint

5. Patients with an impaired elbow function (i.e., stiff or painful elbow or neurological disorder of the upper limb) prior to the injury

6. Retained hardware around the affected elbow

7. History of operations or fractures involving the elbow

8. Patients with rheumatoid arthritis

9. Likely problems, in the judgment of the investigators, with maintaining follow-up (e.g., patients with no fixed address)

10. Insufficient comprehension of the Dutch language to understand a rehabilitation program and other treatment information, which will be judged by the attending physician

Exclusion of a patient because of enrolment in another ongoing drug or surgical intervention trial will be left to the discretion of the attending surgeon on a case-by-case basis.

### Intervention

Reduction can be performed under general, regional, or local anesthesia or without anesthesia, depending upon the preference of the surgeon. The method of choice will be recorded, but not standardized.

Following reduction, the affected arm will be put in either a pressure bandage (e.g., Tubigrip^®^) or a plaster of Paris for three weeks. Both treatment groups will be advised to use a sling; 5-7 days for the functional group, and up to three weeks in the plaster group.

In the functional group, early active movements within the limits of pain are allowed. Patients will be free to select their own physical therapist. Physical therapy is commenced after two days according to a predefined protocol. Patients will be asked to hand over to their physical therapist the following instructions. Exercises will be performed in a supine overhead position with the shoulder flexed at 90°. When coming into the overhead position, the shoulder is held in adduction and neutral to external rotation. The arm is not allowed to cross the midline. This position is controlled by holding the wrist with the healthy hand. In the supine position, with the shoulder in 90° of forward flexion and the forearm maintained in pronation (with the forearm resting on the forehead), gentle active assisted supination and pronation is performed. The second exercise is performed in the same position. The shoulder is placed in 90° of forward flexion and the elbow in 90° or more flexion. The forearm is held in full pronation. Gentle active and active assisted elbow flexion to full range and elbow extension are performed as tolerated and are not to exceed 30°. After three weeks, the sling will be removed, and the supine exercises will be replaced by active and active assisted elbow and forearm motions in the sitting or standing positions.

The plaster group is immobilized for three weeks and after removal of the plaster physical therapy is initiated according to the same protocol as described above.

### Outcome measures

The primary outcome measure is the *Quick-*DASH (Disabilities of the Arm, Shoulder and Hand) score, which reflects both function and pain [15]. The DASH Outcome Measure is a validated 30-item, self-reported questionnaire designed to help describe the disability experienced by people with upper-limb disorders and also to monitor changes in symptoms and function over time [15,16].

The *Quick-*DASH is a shortened version of the DASH Outcome Measure. Instead of 30 items, the Quick-DASH uses 11 items (scored 1-5) to measure physical function and symptoms in people with any or multiple musculoskeletal disorders of the upper limb. The right and left elbow will be assessed separately. At least 10 of the 11 items must be completed for a score to be calculated. The scores will be transformed to a 0-100 scale for easy comparison. A higher score indicates greater disability.

Like the DASH, the *Quick-*DASH contains 2 optional modules to measure symptoms and function in athletes, performing artists and other workers whose jobs require a high degree of physical performance. These optional models are scored separately; each contains four items, scored 1-5. All items must be completed for a score to be calculated.

The secondary outcome measures are:

• Functional outcome (Mayo Elbow Performance Index and Oxford Elbow Score)

• Pain level at both sides (VAS)

• Range of Motion of the elbow joint at both sides

• Rate of secondary interventions

• Rate of complications (secondary dislocation, instability, relaxation)

• Health-related quality of life: SF-36 and EQ-5D

• Radiographic appearance of elbow joint (degenerative changes and heterotopic ossifications)

• Cost

• Cost-effectiveness

The MEPI index is one of the most commonly used physician-based elbow rating systems. This index consists of five parts: pain (with a maximum score of 45 points), ulnohumeral motion (20 points), stability (ten points), the ability to perform five functional tasks (5 × 5 points) and the patient response. If the total score is between 90 and 100 points, it is considered excellent; between 75 and 89 points, good; between 60 and 74 points, fair; and less than 60 points, poor [17].

The Oxford elbow score is a 12-item questionnaire. It is comprised of three one-dimensional domains: elbow function, pain and social-psychological, with each domain comprising 4 items with good measurement properties [18]. This is a validated questionnaire in the UK and was translated to Dutch by the proper translation procedure, which uses the technique of translation and back-translation [19]. Permission for translation and the use of the OES for this study was obtained from Oxford and Isis Outcomes, part of Isis Innovation Limited (website: http://www.isis-innovation.com/)

Pain level will be determined using a 10-point Visual Analog Scale (VAS), in which zero implies no pain and ten implies the worst possible pain.

ROM will be measured on both sides using a goniometer.

Secondary interventions within one year of initial treatment to relieve pain or improve function will be recorded. This includes secondary revision of collateral ligaments and external fixator placement.

Complications within one year of initial treatment will be recorded. These include redislocation, pressure necrosis (plaster group only), post-traumatic dystrophy, and neurologic deficit.

The Short-Form 36 (SF-36) is a validated multi-purpose, short-form health survey with 36 questions that represent eight health domains that are combined into a physical and a mental component scale [20]. The Physical Component Scale (PCS) combines the health domains of physical functioning (PF; ten items), role limitations due to physical health (RP; four items), bodily pain (BP; two items), and general health perceptions (GH; five items). The Mental Component Scale (MCS) combines the health domains of vitality, energy, or fatigue (VT; four items), social functioning (SF; two items), role limitations due to emotional problems (RE; three items), and general mental health (MH; five items). Scores ranging from zero to 100 points are derived for each domain, with lower scores indicating poorer function. These scores will be converted to a norm-based score and compared with the norms for the general population of the United States (1998), in which each scale was scored to have the same average (50 points) and the same standard deviation (ten points).

The EuroQol-5D is a validated questionnaire for health-related quality of life [21,22].

Radiographic appearance (anteroposterior and lateral X-ray at one year): heterotopic ossification will be classified according the classification scheme of Broberg and Morrey as a bone exostosis or as a soft tissue ossification of a ligament, capsule or muscle ("myositis ossificans") [23]; degenerative changes will be classified as grade zero (no change), grade 1 (slight narrowing of the joint space with small osteophytes), grade 2 (moderate narrowing of the joint space, osteophytes and subchondral sclerosis), and grade 3 (severe narrowing of the joint space, large osteophytes, subchondral sclerosis and cystic deformation).

The incremental cost-effectiveness ratio of functional versus plaster treatment will be expressed in a cost-utility ratio, i.e., in terms of cost per QALY. The economic evaluation will be performed from a societal perspective, and will include both health care costs and costs of production losses. Health care costs will include costs of general practice care, medical specialist care, physical therapy, hospitalization, medication, and other costs directly associated with diagnosis, treatment and rehabilitation. Patients will be asked to administer a custom-made questionnaire to register their health care needs and production loss.

In addition to the outcome variables mentioned above, the following data will be collected:

a) Intrinsic variables (baseline data): age, gender, American Society of Anesthesiologists' ASA classification, tobacco consumption, alcohol consumption, comorbidity, social status/household composition, dominant side, and medication use.

b) Injury related variables: affected side, mechanism of injury, and assessment of varus, valgus and posterolateral rotatory instability.

c) Intervention-related variables: reduction delay (i.e., time between dislocation and reduction), time between injury and start of physical therapy, days of sling use, and number of physical therapy sessions

### Study procedures [Table 1]

**Table 1:** Schedule of events

	Screening	Enrollment	Baseline	1 week	3 weeks	6 weeks	3 months	6 months	12 months
				(3-10 d)	(11-28 d)	(4-8 we)	(11-15 we)	(5-7 mo)	(12-14 mo)
**Screening**	X								

**X-ray**	X		X (<24 h post-reduction)	X					X

**Informed Consent**		X							

**Randomization**		X							

**Baseline data**			X						

**Clinical follow-up**				X	X	X	X	X	X

**Revisision surgery**				X	X	X	X	X	X

**Complications**				X	X	X	X	X	X

**Pain (VAS)**				X	X	X	X	X	X

***Quick-*DASH**						X	X	X	X

**MEPI**						X	X	X	X

**Oxford Elbow Score**						X	X	X	X

**SF-36**						X	X	X	X

**EQ-5D**						X	X	X	X

**Health care consumption**						X	X	X	X

**ROM**						X	X	X	X

**Early withdrawal**				*	*	*	*	*	*

*, only if applicable

Clinical assessments will occur at the time of admission (baseline), one week (3-10-day window), three weeks (11-28-day window), six weeks (4-8-week window), three months (11-15-week window), six months (5-7-month window), and 12 months (12-14-month window) after start of treatment.

At each FU visit, the research coordinator or research assistant will ascertain patient status (i.e., secondary interventions, adverse events/complications, deaths) and will verify information within medical records.

At each FU visit, the patients will be asked to indicate the pain level on a VAS.

At each visit from six weeks onwards, the ROM of the elbow will be measured using a goniometer by a doctor blinded for the treatment of the dislocation. This will be used to calculate the MEPI index. In addition, patients will be asked to complete the questionnaires relating to disability (*Quick-*DASH score including optional modules, Oxford Elbow Score), health-related quality of life (SF-36, EQ-5D), and healthcare consumption.

Plain X-rays of the elbow will be made at the time of presentation in the hospital (baseline), post-reduction, and at the follow-up visit after one week and one year. The X-ray at 12 months will be taken in order to determine the amount and location of heterotopic ossification and the grade of degenerative joint changes. This is common practice in this type of patient. At the last visit, the surgeon will document any surgery that may be planned for the patient.

### Sample size calculation

Calculation of the required sample size is based upon the assumption that the mean *Quick-*DASH will be 12.5 in plaster treated patients and five in the functional group, assuming a standard deviation of 15 for the plaster group and 7.5 for the functional group [7]. A 2-sided test with an α level of 0.05 and a β level of 0.2 requires 41 patients in each group. Anticipating a dropout rate of 20% loss to follow-up a sample size of 50 patients in each arm is required.

### Statistical analysis

Data will be analyzed using the PASW Statistics version 18.0.1 or higher (SPSS, Chicago, Illinois, USA). Normality of continuous data will be tested with the Shapiro-Wilk and Kolmogorov-Smirnov test and by inspecting the frequency distributions (histograms). The homogeneity of variances will be tested using the Levene's test.

The analysis will be performed on an intention to treat basis. Patients with protocol violations will be followed up, and data will be recorded. Data will be analyzed with and without inclusion of patients with protocol violation.

Descriptive analysis will be performed to report baseline characteristics (intrinsic variables and injury-related variables) in both treatment groups. For continuous data (e.g., age, Quick-DASH score at baseline) mean ± SD (parametric data) or medians and percentiles (non-parametric data) will be calculated. For categorical data (e.g., gender, ASA grade, alcohol and tobacco consumption, dominant and affected side) frequencies will be calculated.

The mean difference between the mean *Quick-*DASH scores of the functional group and the plaster group will be tested. Univariate analysis will be performed to test the difference in the primary and secondary outcome measures between the functional and the plaster groups. Continuous data will be tested using a Student's T-test (parametric data) or a Mann Whitney *U*-test (non-parametric data). Chi-square analysis will be used for statistical testing of categorical data. A p-value <0.05 will be taken as the threshold of statistical significance.

A multivariable linear regression analysis will be performed to model the relationship between different covariates and the *Quick-*DASH score. Intrinsic and injury-related variables that display a p-value <0.5 in the univariate analyses will be added as a covariate.

### Ethical considerations

The study will be conducted according to the principles of the Declaration of Helsinki (59th World Medical Association General Assembly, Seoul, October 2008) and in accordance with the Medical Research Involving Human Subjects Act (WMO).

The Medical Ethics Committee Erasmus MC (Rotterdam, The Netherlands) acts as central ethics committee for this trial (reference number MEC-2009-239; NL28124.078.09). Approval has been obtained from the local Medical Ethics Committees in all participating centers. An information letter notifying the patients' participation will be sent to their general practitioners, unless a patient does not agree with this.

The Medical Ethics Committee Erasmus MC has given dispensation from the statutory obligation to provide insurance for subjects participating in medical research (article 7, subsection 6 of the WMO and Medical Research (Human Subjects) Compulsory Insurance Decree of 23 June 2003). The reason for this dispensation is that participation in this study is without risks.

## Discussion

The FuncSiE trial will compare management of simple elbow dislocations by early functional treatment with treatment by plaster immobilization. Early functional treatment may lead to a better ROM and prevent elbow stiffness. To date no RCT for the management of simple elbow dislocation has been performed with a sample size of 100 patients. Inclusion of patients has been started August 26, 2009 and the expectation is to include 8 patients per month. With a follow-up of one year the presentation of data will be expected in the beginning of 2012.

## Abbreviations

ASA: American Society of Anesthesiologists; BP: Bodily Pain; CONSORT: CONsolidated Standards of Reporting Trial; DASH: Disabilities of the Arm: Shoulder and Hand score; ED: Emergency Department; EQ-5D: EuroQol-5D; GH: General Health perception; HR-QoL: Health-related Quality of Life; MCS: Mental Component Scale; MEPI: Mayo Elbow Performance Index; MH: general Mental Health; NTR: Netherlands Trial Registry (in Dutch: Nederlands Trial Register); PCS: Physical Component Scale; PF: physical functioning; QALY: Quality-Adjusted Life Years; QoL: Quality of Life; RCT: Randomized Controlled Trial; RE: Role limitations due to Emotional problems; ROM: Range Of Motion; RP: role limitations due to physical health; SF: Social Functioning; SF-36: Short Form 36; SPSS: Statistical Package for the Social Sciences; VAS: Visual Analog Scale; VT: vitality, energy, or fatigue.

## Competing interests

The authors declare that they have no competing interests.

## Authors' contributions

JDH, DDH, WET, EMMVL, and NWLS developed the trial and drafter the manuscript. NWLS will act as trial principal investigator. SP assisted in the design of the healthcare consumption questionnaire and will perform the health economic analyses. WET, EMMVL and NWLS will perform statistical analysis of the trial data. DDH, GITI, RSB, MWGAB, MMMB, MRDV, BJD, DE, RH, SAGM, JWRM, KJP, WHR, GRR, IBS, MAS, JBS, SS, JGHVDB, HGWMVDM, TPHVT, ABVV, EJMMV, JPAMV, MW, WJW, PP, and NWLS will participate in patient inclusion and assessment. All authors have read and approved the final manuscript.

## Specified notice

Oxford Elbow Score^© ^Isis Innovation Limited, 2008. All rights reserved. The authors, being Professor Ray Fitzpatrick and Dr Jill Dawson, have asserted their moral rights.

## Pre-publication history

The pre-publication history for this paper can be accessed here:

http://www.biomedcentral.com/1471-2474/11/263/prepub
